# The Bright Side of Hematopoiesis: Regulatory Roles of ARID3a/Bright in Human and Mouse Hematopoiesis

**DOI:** 10.3389/fimmu.2014.00113

**Published:** 2014-03-19

**Authors:** Michelle L. Ratliff, Troy D. Templeton, Julie M. Ward, Carol F. Webb

**Affiliations:** ^1^Immunobiology and Cancer Research, Oklahoma Medical Research Foundation, Oklahoma City, OK, USA; ^2^Department of Cell Biology, University of Oklahoma Health Sciences Center, Oklahoma City, OK, USA; ^3^Department of Microbiology and Immunology, University of Oklahoma Health Sciences Center, Oklahoma City, OK, USA

**Keywords:** Bright, ARID3a, hematopoietic regulation, B cell development, gene regulation

## Abstract

ARID3a/Bright is a DNA-binding protein that was originally discovered for its ability to increase immunoglobulin transcription in antigen-activated B cells. It interacts with DNA as a dimer through its ARID, or A/T-rich interacting domain. In association with other proteins, ARID3a increased transcription of the immunoglobulin heavy chain and led to improved chromatin accessibility of the heavy chain enhancer. Constitutive expression of ARID3a in B lineage cells resulted in autoantibody production, suggesting its regulation is important. Abnormal ARID3a expression has also been associated with increased proliferative capacity and malignancy. Roles for ARID3a in addition to interactions with the immunoglobulin locus were suggested by transgenic and knockout mouse models. Over-expression of ARID3a resulted in skewing of mature B cell subsets and altered gene expression patterns of follicular B cells, whereas loss of function resulted in loss of B1 lineage B cells and defects in hematopoiesis. More recent studies showed that loss of ARID3a in adult somatic cells promoted developmental plasticity, alterations in gene expression patterns, and lineage fate decisions. Together, these data suggest new regulatory roles for ARID3a. The genes influenced by ARID3a are likely to play pivotal roles in lineage decisions, highlighting the importance of this understudied transcription factor.

Bright (B cell regulator of immunoglobulin heavy chain transcription) is a 70 kDa DNA-binding protein first characterized in the mouse as a component of a protein complex associated with increased transcription of the immunoglobulin heavy chain (IgH) locus in activated B lymphocytes (1–3). *Bright, also known as DRIL1, E2FBP1, or ARID3a* (the designation for the human ortholog, hereafter referred to as Bright), is a member of the A + T rich interaction domain (ARID) protein family, many of which have been shown recently to have epigenetic regulatory functions [reviewed in Ref. (4–6)]. These proteins bind to A + T rich DNA sequences and are typically members of larger chromatin modulatory complexes. Bright, and other ARID3 family members, require dimerization for DNA-binding activity and contain an extended DNA-binding domain that confers increased DNA sequence specificity to these proteins compared to other ARID family members (7–9). Although Bright was the first member of this family identified in mammalian cells, its functions have only begun to be elucidated. Previously, Bright expression in adult, mouse, and human cells was thought to be largely restricted to B lymphocyte lineage cells. However, more recently, we and others have shown that Bright plays important regulatory functions in early hematopoiesis. Although Bright expression is restricted in adults, it is more widely expressed in the embryo/fetus and plays important regulatory roles in embryonic stem cell differentiation (10). These data also highlight novel roles for Bright in gene repression. This article will emphasize the regulatory roles of Bright in hematopoiesis and will summarize new contributions pertaining to its regulatory capacity in those and other cell types.

## Bright and HSCs

**Figure 1:**
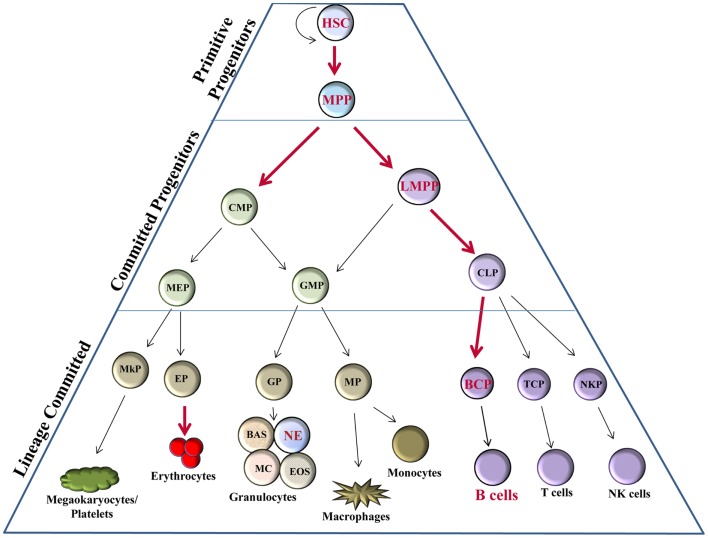
**Bright expression in hematopoiesis**. Hematopoietic progenitor populations [as described (11)] indicate stages, which express Bright/ARID3a (red font) versus those not known to express Bright (black font). Thick red arrows indicate stages of developmental progression for which Bright function is important. HSC, hematopoietic stem cell; MPP, multipotent progenitor; LMPP, lymphoid-primed multipotent progenitor; CMP, common myeloid progenitor; CLP, common lymphoid progenitor; GMP, granulocyte-macrophage progenitor; MEP, megakaryocyte–erythrocyte progenitor; BCP, B cell progenitor; TCP, T cell progenitor; NKP, NK cell progenitor; MP, macrophage progenitor; GP, granulocyte progenitor; EP, erythrocyte progenitor; MkP, megakaryocyte progenitor; BAS, basophils; NE, neutrophils; MC, mast cells; EOS, eosinophils.

From a historical perspective, the majority of studies involving Bright have aimed at understanding its roles in B lymphocytes. However, recent evidence suggests Bright may play an even broader role in the development of hematopoietic lineage cells. Hematopoietic stem cells (HSCs) have the capacity to self-renew or to differentiate into other precursors that will eventually produce all mature blood cell types. Differentiation of hematopoietic progenitors occurs primarily along three pathways: erythroid, myeloid, and lymphoid lineages (Figure 1). An intricate network of transcription factors contribute to HSC fate decisions, with more than 20 transcription factors implicated in the development of various hematopoietic subpopulations [reviewed in Ref. (11)], such as growth factor independence 1 (Gfi1), E2A, and Ikaros family zinc-finger protein 1 (Ikaros) in lymphoid lineage regulation; CCAAT-enhancer binding protein alpha (C/EBPα), GATA1, and PU.1 for myeloid lineage decisions [reviewed in Ref. (11–13)]. Bright is expressed in HSCs in both mouse and man [(14–16); and our unpublished data] and appears to be required for development of several early progenitor subsets including multipotent progenitors (MPPs) and lymphoid-primed MPP (LMPP) (Figure 1). Therefore, Bright contributes to early progenitor ontogeny, which may ultimately affect the development of multiple lineages.

Bright knockout mice die between E11.5 and E13.5 as a result of defects in erythroid lineage differentiation (16). Bright knockout embryos have severe pallor and show fewer mature erythrocytes by flow cytometry. Embryonic death in Bright-deficient mice coincides with the shift from primitive hematopoiesis in the yolk sac to definitive hematopoiesis in the fetal liver. Numbers of fetal liver lin^−^cKit^hi^Sca1^+^CD150^+^CD48^−^ HSCs in these embryos were reduced by >90%, while LSKs (Lin^−^Sca1^+^cKit^+^ cells that include HSC and MPP populations) were decreased in Bright deficient versus wild-type littermate controls by 80% (16). Numbers of common myeloid progenitors (CMPs) and common lymphoid progenitors (CLPs) were also decreased in Bright knockout fetal livers, but to a lesser degree. This suggests that Bright may have greater effects in HSCs than in later precursor subsets. Bright knockout fetal liver cells were also impaired in their ability to generate erythroblast, erythromyeloid, and B lymphocyte colonies in *in vitro* methylcellulose cultures compared to wild-type cells (16). Therefore, Bright may also contribute to the expansion and development of these hematopoietic cells. Importantly, these data confirm the importance of Bright for normal erythroid differentiation.

Rare Bright knockout mice (<1%) survived to adulthood for unknown reasons. These adult Bright-deficient mice exhibited reduced numbers of hematopoietic precursors in the bone marrow, including LSK, CMP, and CLP subsets, but to a lesser degree than was observed in Bright null embryos (16). Although adult Bright-deficient mice showed decreased numbers of several early B cell subsets, interestingly, no deficiencies in T cell or erythrocyte development were observed (16). The importance of Bright in other hematopoietic lineages has not been fully explored; however, recent data from The Immunological Genome Project suggest Bright transcripts are expressed in mouse neutrophils where its function is unclear (17). Currently, our data from knockout mouse models suggest that the function of Bright in adult bone marrow may be more critical for the development of B lymphocytes and early precursor subpopulations of those cells than for other hematopoietic cell types.

## Bright Side of B Cells

**Figure 2:**
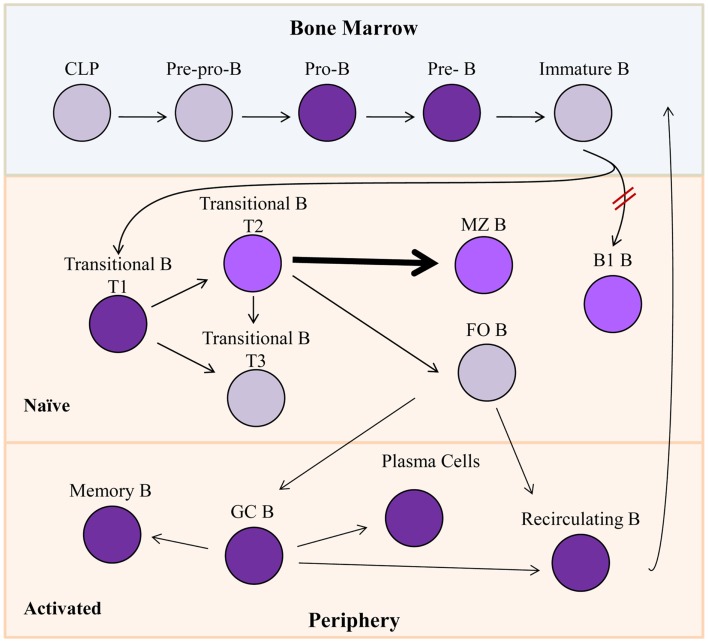
**Bright expression during B lymphopoiesis**. Gray cells indicate B lineage subsets not known to express Bright. Purple subsets express Bright, while lighter shades (T2, MZ B, and B1 B) represent slightly lower levels of Bright expression. Bright is required for development of B1 B cells indicated by red hash marks. Bolded arrow indicates developmental skewing caused by Bright over-expression. CLP, common lymphoid progenitor; MZ B, marginal zone B cells; FO B, follicular B cell; GC B, germinal center B cells.

Common lymphoid progenitors give rise to early subpopulations of precursor B cells (Figure 2), which eventually develop through multiple differentiation states in both the bone marrow and the periphery to result in fully differentiated, mature B cells. Pro-B cells express RAG1/2 gene products, and rearrange IgH gene segments (18, 19), and in man, they are the first B cell progenitors to transcribe Bright (14). Bright expression occurs in both mouse and human subsets of pre-B cells, transitional B cells, activated and memory B cells, and plasma cells (14, 15) (Figure 2). However, the majority of resting, naïve, and mature, peripheral B lymphocytes do not express Bright as evidenced by the absence of Bright transcripts in the majority of circulating blood cells in man, and in follicular spleen cells in the mouse (14, 15). Both human and mouse innate-like B cells, represented by peritoneal cavity B1 B lymphocytes and splenic marginal zone (MZ) B lymphocytes in mice, and by B1-like and MZ-like cells in human peripheral blood [reviewed in Ref. (20, 21)], express low levels of Bright [(22); unpublished data]. Thus, Bright expression is tightly regulated at the level of transcription throughout B cell differentiation.

Transgenic mice expressing a dominant negative form of Bright from the CD19 pan-B cell promoter were generated by introduction of point mutations in the DNA-binding domain to test the function of Bright within B lineage cells (8, 22). These transgenic mice had slightly decreased numbers of mature B cells, decreased serum IgM levels, and defective responses against *Streptococci* (22), phenotypes similar to those observed in Bruton’s tyrosine kinase (Btk) deficient mice [(23), reviewed in Ref. (24, 25)]. Although these mice developed mature B1 B cells, a major source of IgM in the mouse, those cells were functionally defective in their ability to secrete immunoglobulin (22). Furthermore, the few total Bright knockout mice that survived to adulthood lacked B1 B cells (16), suggesting Bright is important both for development and function of those mature B cells. Adult Bright-deficient mice also showed impaired T cell-dependent IgG_1_ production due to defects in class switch recombination (16). Thus, Bright contributes to select functions in mature B cells.

Forced expression of native Bright throughout the B cell lineage suggested that regulation of Bright is critical for normal development of MZ and follicular B cells. Transgenic FVB/N mice constitutively over-expressing Bright from the CD19 promoter exhibited significant increase in immature transitional B and MZ B cells relative to the other splenic B cell populations (26). On the C57BL/6 background, over-expression of Bright also resulted in decreased numbers of follicular (FO) B cells (27). The FO B cells that were present in those mice displayed transcript levels of genes previously shown to be differentially expressed in FO versus MZ B cells (28) that were similar to those observed in MZ B cells. Chimeras generated from mixtures of transgenic and wild-type bone marrow cells also showed preferential development of MZ versus FO B cells (27), suggesting that constitutive Bright expression contributes preferentially to MZ versus FO B cell development.

Bright expression can be induced in mature resting B cells through a number of activating signals, including stimulation with lipopolysaccharide (LPS), CD40 ligand, interleukin-5 (IL-5) plus specific antigen, and with agonistic monoclonal antibodies against CD38 or RP105 (2, 3, 15). Additionally, Epstein–Barr Virus (EBV) infection also activates Bright expression in human B cells (14). Although the function of Bright in B lymphocytes was thought to be exerted primarily in the nucleus via interactions with A + T-rich DNA sequences, a very small percentage of Bright was discovered to be palmitoylated and diverted to lipid rafts (29). *In vitro* studies demonstrated Bright localization to these rafts increased the signaling threshold of the B cell receptor (BCR). Upon effective activation, Bright was released from the lipid rafts via SUMOylation. Interestingly, Btk remained unphosphorylated when Bright was present, delineating a putative role for Bright in BCR signaling (29). However, FO B cells from transgenic mice with forced expression of Bright did not have elevated levels of Bright in lipid rafts, in contrary to transitional and MZ B cell populations, nor did they have alterations in their ability to flux calcium through the BCR. Together, these data suggested other cell type-specific factors may contribute to Bright-mediated effects through the BCR (27).

## Gene Targets for Bright

It was originally observed that stimulation of an antigen-specific mouse B cell line with antigen and IL-5 resulted in an increase in immunoglobulin (μ) heavy chain transcription. Further analyses identified two discrete A + T rich elements within the V1 S107 variable heavy chain promoter were bound by a protein complex later identified to contain Bright (3). Intriguingly, further analyses showed potential Bright-binding motifs in about half of the murine V_H_ promoters, and binding sites were not restricted to specific V region families (30, 31). Similarly, only a subset of human V_H_ promoters had binding sites for Bright (32). These data suggest that Bright may preferentially affect transcription of a subset of IgH genes.

**Figure 3:**
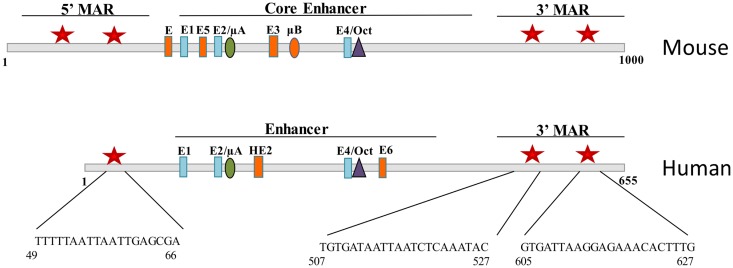
**Bright binds to sequences flanking the mouse and human IgH enhancer**. Schematic diagrams of fragments including the Eμ core enhancer show binding site for E proteins, Ets proteins (μA, μB, HE2), and Oct transcription factors. Matrix association regions (MARs) identified by MAR-binding assays are indicated by red stars on either side of the core (1). Orange enhancer elements denote those regions that are not conserved between species.

In the mouse, the intronic enhancer of the IgH gene is flanked by 5′ and 3′ A + T rich regions called matrix associated regions (MARs) that act to tether DNA to the nuclear matrix. Promoter binding sites for Bright were shown to have MAR activity (1), and conversely, Bright was shown to bind to the MARs of the intronic IgH enhancer in the mouse (7). Mouse and human IgH enhancers are similar, but not identical, showing some differences in binding sites for several transcription factors (Figure 3). Bright-binding sites flank both the mouse and human enhancer core sequences. Although Bright did not directly activate transcription of the IgH enhancer regions *in vitro*, in contrast to promoter fragments that bound Bright (33), MAR regions bound by Bright have been proposed to play important roles establishing chromatin domains important for expression. Indeed, the intronic enhancer region in the mouse locus has been shown to be important for chromatin remodeling, and binding of Bright to sites flanking that region correlated with increased enhancer accessibility (34). Bright can also form tetramers and was found to enhance DNA-bending, suggesting it may also contribute to higher order structures linking the intronic enhancer to specific V_H_ region promoters (35). Therefore, we speculate that Bright, like other members of the ARID family, may contribute to epigenetic regulation of the IgH locus.

Recently, additional gene targets for Bright have been identified. Bright was shown to bind to the core promoter sequence of the EBV C promoter (Cp) where it interacts with E2F-1 and Oct-2, and to the family of repeats (FR) region at the latent origin of plasmid replication (oriP) in the EBV plasmid (36). Cp regulates expression of genes required for B cell proliferation in latent EBV infections. The FR regions are upstream of Cp, functioning as an essential enhancer to this promoter, and this interaction is mediated by Epstein–Barr nuclear antigen 1 (EBNA1) (37). Together, these interactions lead to the initiation of transcription of EBV latency proteins (36), suggesting that Bright contributes to maintenance of EBV in certain B cell subsets.

Additional putative gene targets for Bright/ARID3a have been identified as part of the ENCODE project, and that database now lists a number of potential gene targets for Bright/ARID3a in human cell lines (38). One target of particular interest is *Oct4*. We found that Bright binds to the *Oct4* promoter and acts to repress its transcription in mouse embryonic fibroblasts (39). These data are the first to indicate Bright can have repressive as well as activating functions, such as those described for the IgH locus. Further, the *Drosophila* ortholog of Bright, *Dril1*, recruits *Groucho* to *Dorsal* where it is also associated with strong repressive potential (6). It is likely that Bright levels will affect multiple gene targets in hematopoietic cells, where it will be important to consider that it may also act to suppress gene expression.

## Co-Regulatory and Interacting Proteins

Dimerization of Bright was required for binding to the IgH locus in mobility shift assays (8). Earlier antibody supershift assays were the first to suggest that the Bright complex might also contain additional proteins, potentially including topoisomerase II (40). The S107 V_H_ gene, containing the prototypic Bright-binding site, was required for immune responses against phosphorylcholine, a response deficient in mice lacking Btk [Ref. (23), reviewed in Ref. (24, 25)]. These data led to experiments demonstrating that Btk interacted directly with Bright, and that its kinase activity was required for Bright-associated transactivation of the IgH promoter (41). Further studies demonstrated that the transcription factor TFII-I, a Btk target [reviewed in Ref. (42)], also bound to Bright through the helix-turn helix domain at the carboxyl end of Bright, and that this interaction was also required for activation of the immunoglobulin locus (33). TFII-I is ubiquitously expressed in multiple isoforms and functions as a transcription factor for basally expressed genes and a transcription activator for upstream protein complexes [reviewed in Ref. (42)]. This suggests that Bright may act as a DNA-binding component to recruit or tether other transcription activating proteins to specific promoter sites.

A truncated form of human Bright/ARID3a was cloned and identified by others from embryonal carcinoma cells as an E2F-binding protein, E2FBP1 (43). These studies showed interactions of Bright and E2F-1, a pRB-controlled protein important for cell cycle regulation, and linked Bright function to cell cycle regulated functions. Functional screens for products that rescued Ras-induced senescence in mouse embryonic fibroblasts also identified Bright (hDril1) as a candidate protein (44). Those studies also linked Dril1/Bright functions to proliferation and pRB-mediated pathways; however, results using human cells did not mirror those observed in mouse embryonic fibroblasts. In other model systems that required p53, Bright over-expression induced G1 arrest by activating p21 in response to DNA damage in human osteosarcoma cells (45). In this and other studies, ARID3a was found to be a direct downstream target of p53, and a co-regulator with p53 of other gene targets (45, 46). The multiple roles played by Bright/ARID3a during the cell cycle in different cell types highlight the necessity to use caution in interpreting data that may be of a complex nature and the result of interactions with different intracellular mediators. Interpretation of studies involving over-expression of Bright may be further complicated, as levels of Bright within cells of the same lineage appear to be tightly regulated as a consequence of the differentiation state (14). Indeed, SUMOylation of Bright was reported to impair interactions with E2F-1 while promoting transcriptional activation of myeloid lineage-specific genes in HSC populations (47), indicating further complexities that may exist in some cell types due to post-translational modifications of Bright and/or the proteins with which it may interact.

Bright has also been shown to interact with several components of promyelocytic leukemia nuclear bodies (PML NBs), including the ubiquitously expressed protein SP100 and the lymphoid-restricted homolog, LYSp100B (48). Bright was found to colocalize with Sp100 in PML NBs and was found to repress Bright transactivation functions, while LYSp100 strongly stimulated Bright transactivation of the IgH locus. These data support functions for Bright in higher order chromatin topology and epigenetic regulation. Other studies suggest that levels of Bright/E2FBP1 are important for maintenance of PML NBs and cell viability (49, 50). Some viral proteins, including those from human herpes simplex viruses, also disrupt PML NBs and show linked regulatory effects with Bright/E2FBP1 (51). Clearly, it will be important to understand further consequences of Bright functions in these important nuclear structures.

Finally, as we continue to see examples of transcription factors that interact to form large chromatin modulatory complexes or interactomes that regulate large sets of genes involved in specific cellular processes, it is likely that we will identify new protein partners for Bright. Recent findings suggest Bright/ARID3a is one of a number of genes induced by gamma-interferon in Th1 cells, a T helper cell subset previously unknown to express Bright (52). These findings emphasize again the likelihood that Bright may play distinct regulatory roles in different types of cells.

## Regulation of Bright

Id1, a member of a family of three proteins described to be negative regulators of E2A proteins (53, 54), was also shown to interact with Bright/Dril1 in human fetal lung fibroblasts and in lung fibroblasts from patients with idiopathic fibrosis (55). As with other factors inhibited by Id proteins, Id1 formed a complex with Bright that abrogated its DNA-binding activity. In fibrotic lung tissues, Bright was expressed abundantly as a consequence of TGF-β signaling (55). Lending further support that Bright may be a downstream mediator of TGF-β signaling, the Bright ortholog in *Xenopus* was required for normal development of mesoderm in embryos, through SMAD2-dependent TGFβ pathways (56). Furthermore, our lab has observed a link between levels of Bright in human B lymphocytes and expression of TGF-β pathway associated genes (our unpublished data), additionally supporting associations between TGF-β signaling and Bright induction. Id proteins, important for regulation of Bright in lung tissues (55), have also been shown to be important in lineage decisions and in directing B cells toward MZ versus FO B cell phenotypes (57, 58). As mentioned previously, we demonstrated that levels of Bright contribute to those same B cell lineage decisions in transgenic mouse models (27). Therefore, we speculate that Id proteins may also regulate Bright during B cell development.

Very little is known regarding Bright regulation. In B lymphocyte lineage cells, Bright is tightly regulated during differentiation at the level of transcription (14). An important microRNA family regulating transcript levels during hematopoiesis is miRNA125. This microRNA family consists of three members that function at different stages of this process. Myeloid lineage fate decisions have been reported to be regulated by miR125b, pushing granulocyte-macrophage progenitors for the myeloid lineage toward macrophage differentiation (59). Previous studies characterized expression of the miR125 family of micro RNAs in human B lymphocytes at various stages of differentiation, showing members of this family were differentially expressed according to the maturation state of the cells (60). In addition, these studies indicated that over-expression of miR125b inhibited B cell differentiation and affected survival of myeloma cells. Although it is unclear whether some of these effects may be mediated through suppression of Bright, others have shown that Bright is a direct target of miR125b in B cell progenitors (61). Expression of miR125b in human pre-BI cells increased their proliferation in culture. Similar responses were observed in B cell acute lymphocytic leukemias (B-ALL), where these effects were mediated through suppression of ARID3a (61). Suppression of ARID3a in those cells also resulted in decreased apoptosis in a p53-independent fashion. Interestingly, increased expression of miR125b did not block *in vitro* pre-B differentiation (61). Thus, Bright functions likely differ according to the maturation state of the B cell.

## Implications in Health and Disease

Because Bright was first identified in B lymphocytes, its functions have been better elucidated in those cells. Over-expression of Bright in mouse B lineage cells increased production of autoantibodies with anti-nuclear antigen (ANA) specificities (26, 27). These antibodies formed immunoglobulin deposits in the kidney glomeruli, although this did not affect kidney function nor did mice display autoimmune phenotypes that threatened mortality. These data imply that Bright over-expression in B lineage cells may predispose those cells toward autoreactive phenotypes, either by expanding B cells with autoimmune phenotypes or by allowing their escape from important tolerance checkpoints. Increased numbers of T1 transitional B cells and MZ B cells have been implicated in autoimmune disease in both mouse and human and both of these B lineage subsets express Bright (26, 27). ANA production is a defining characteristic of autoimmune patients with systemic lupus erythematosus (SLE), and we found that those patients also show increased numbers of Bright^+^ peripheral blood B lineage cells that are associated with increased disease activity (our unpublished results). Several studies have linked EBV and SLE, with nearly all pediatric and adult SLE showing EBV infection [reviewed in Ref. (62)]. Intriguingly, Bright is induced in human B cells upon infection with EBV (14), and EBV requires Bright for maintenance of latency genes (36). In addition, many miRNAs have been noted to be differentially expressed in SLE patients versus healthy controls, and miR125a, a member of the family responsible for down-regulation of Bright activity in B cell progenitors (61), was described as being down-regulated in lupus lymphocytes [reviewed in Ref. (63)]. Together, these studies highlight the importance for future mechanistic studies to explore the links between Bright expression and ANA production in SLE patients and their relationship to epigenetic changes implicated in SLE pathology (64).

Bright dysregulation has also been implicated in several types of malignancies, including those derived from hematopoietic lineage cells. Analyses of diffuse large B cell lymphomas (DLBCL) over a decade ago identified two distinct subtypes of those malignancies with different survival advantages, the activated B-like (ABC) and germinal center B-like (GCB) DLBCL by gene expression profiling, which indicated differential ARID3a expression in the two subsets (65). More recent large scale comparative analyses of gene expression patterns in >2000 cases of DLBCL identified Bright/ARID3a as a member of a family of transcription factor signature genes consistently associated with ABC DLBCL, suggesting it might be a useful marker for identification of this subset of lymphoma (66). To the contrary, in the pre-B cell malignancy, B-ALL, Bright expression was down-regulated compared to levels found in healthy pre-B cells as a consequence of 30- to 600-fold higher expression of miR125b, resulting in the oncogenic properties of increased proliferation and cell survival (61). These data suggest that dysregulated Bright/ARID3a levels may contribute to malignancy. This may not be surprising in light of associations between Bright, p53, and other critical cell cycle mediators described above. In keeping with the multiple advances describing additional cell types expressing ARID3a, recent studies also indicate that high levels of Bright/ARID3a may distinguish colorectal carcinomas with good prognosis and a more differentiated phenotype (67).

Interestingly, a B-ALL patient sample with down-regulated levels of Bright was reported to have significant upregulation of pluripotent factors (61). We previously showed that loss of Bright expression in mouse B lineage cells up-regulated pluripotency gene expression and promoted developmental plasticity, giving rise to cells that resembled induced pluripotent stem cells (10). Many types of malignancies are proposed to contain adult stem cell populations that contribute to their oncogenic potential [reviewed in Ref. (68)]. Stem cells, as a consequence of their self-renewal properties, express many genes commonly dysregulated in oncogenesis. Our recent studies show that Bright knockout mouse embryonic fibroblasts can spontaneously form stem cell-like colonies and the key pluripotency factor, Oct4, is repressed as a consequence of Bright function (39). The ability to reprogram cells from multiple sources, including hematopoietic lineage cells, by manipulating expression of Oct4 and other pluripotency-related transcripts has tremendous potential for regenerative medicine (69). We hypothesize that directed manipulation of Bright levels will also have useful applications in regeneration of some cell types. The next decade is full of Bright promise.

## Conflict of Interest Statement

The authors declare that the research was conducted in the absence of any commercial or financial relationships that could be construed as a potential conflict of interest.
